# Pharmacological Management of Borderline Personality Disorder: The Role of Antipsychotics

**DOI:** 10.1192/j.eurpsy.2026.11913

**Published:** 2026-07-31

**Authors:** A. Rami

**Affiliations:** Razi Hospital, Manouba, Tunisia

## Abstract

**Introduction:**

Borderline Personality Disorder (BPD) is a complex and heterogeneous psychiatric condition characterized by emotional instability, impulsivity, disturbed self-image, and recurrent suicidal behaviors. Despite the central role of psychotherapy,particularly dialectical behavior therapy (DBT),in its management, pharmacotherapy remains widely used to target specific symptom domains. Among psychotropic agents, **antipsychotics** are frequently prescribed, although their use in BPD is **off-label** and often debated.

**Objectives:**

This literature review aims to synthesize current evidence regarding the **efficacy, safety, and clinical rationale** for prescribing antipsychotics in the treatment of borderline personality disorder.

**Methods:**

A systematic search of the scientific literature published between 2010 and 2025 was conducted using PubMed, ScienceDirect, and PsycINFO. Randomized controlled trials, meta-analyses, and clinical guidelines were analyzed, focusing on both **typical** and **atypical antipsychotics** used in adult BPD populations.

**Results:**

Evidence suggests that **second-generation antipsychotics**—such as **olanzapine, aripiprazole, quetiapine, and risperidone**—can produce **modest but clinically meaningful improvements** in specific symptom clusters, including **impulsivity, anger, affective dysregulation, and cognitive-perceptual distortions**. However, effects on core features such as interpersonal instability and chronic emptiness remain limited. Long-term efficacy is uncertain, and **treatment discontinuation rates are high** due to side effects like weight gain, metabolic disturbances, and sedation. Guidelines (e.g., NICE, APA, HAS) consistently recommend **restricting antipsychotic use to short-term, symptom-targeted adjunctive therapy**, in combination with structured psychotherapy.

**Conclusions:**

Antipsychotics can be **useful adjuncts** for managing acute symptom exacerbations in borderline personality disorder, particularly when impulsivity or transient psychotic features are prominent. Nevertheless, their **off-label status**, **modest efficacy**, and **adverse-effect profile** warrant cautious and individualized use. Future research should clarify optimal treatment duration, dosing strategies, and predictive markers of pharmacological response, while reinforcing psychotherapy as the **cornerstone of care** in BPD.

**Disclosure of Interest:**

None Declared

